# Theoretical and Experimental Optimization of the Graft Density of Functionalized Anti-Biofouling Surfaces by Cationic Brushes

**DOI:** 10.3390/membranes10120431

**Published:** 2020-12-17

**Authors:** Yijie Ren, Hongxia Zhou, Jin Lu, Sicheng Huang, Haomiao Zhu, Li Li

**Affiliations:** 1 National and Local Joint Engineering Research Center of Biomedical Functional Materials, Nanjing Normal University, No. 1 Wenyuan Road, Nanjing 210023, China; njnu17@163.com (Y.R.); 171102039@njnu.edu.cn (H.Z.); 131102049@njnu.edu.cn (J.L.); lllsnjnu@sina.com (S.H.); 2 School of Chemistry and Materials Science, Nanjing Normal University, No. 1 Wenyuan Road, Nanjing 210023, China; 3 Jiangsu Collaborative Innovation Center of Biomedical Functional Materials, No. 1 Wenyuan Road, Nanjing 210023, China; 4 Jiangsu Key Laboratory of Biofunctional Materials, Jiangsu Engineering Research Center for Biomedical Function Materials, No. 1 Wenyuan Road, Nanjing 210023, China

**Keywords:** polyurethane, cationic brushes, graft density, hemocompatibility, anti-biofouling, catheter

## Abstract

Diseases and complications related to catheter materials are severe problems in biomedical material applications, increasing the infection risk and medical expenses. Therefore, there is an enormous demand for catheter materials with antibacterial and antifouling properties. Considering this, in this work, we developed an approach of constructing antibacterial surfaces on polyurethane (PU) via surface-initiated atom transfer radical polymerization (SI-ATRP). A variety of cationic polymers were grafted on PU. The biocompatibility and antifouling properties of all resulting materials were evaluated and compared. We also used a theoretical algorithm to investigate the anticoagulant mechanism of our PU-based grafts. The hemocompatibility and anti-biofouling performance improved at a 86–112 μg/cm^2^ grafting density. The theoretical simulation demonstrated that the in vivo anti-fouling performance and optimal biocompatibility of our PU-based materials could be achieved at a 20% grafting degree. We also discuss the mechanism responsible for the hemocompatibility of the cationic brushes fabricated in this work. The results reported in this paper provide insights and novel ideas on material design for applications related to medical catheters.

## 1. Introduction

Catheter-related diseases and complications are serious pathogenic problems which increase the infection risk and medical expenses [1,2]. Catheter-related bloodstream infections (CRBSIs) and deep venous thrombosis (DVT) are among the worst drawbacks [3,4]. Bacteria and non-specific proteins impregnably adhere to the hydrophobic catheter surfaces, which often leads to coagulation and bacterial infection after prolonged contact with blood [5,6,7]. A patient with such complications relies on heparin and antibiotic injections. However, heparin remains in blood vessels for prolonged periods [8]. Therefore, the development of improved catheter materials capable of minimizing these problems has become more and more critical.

A thin aqueous layer adsorbed on a material surface can reduce its protein adsorption by changing the protein orientation and conformation [9,10,11]. Modeling results published by Cormack et al. [12] confirmed that the primary factor responsible for protein adsorption is the double layer at the water/material interface. Protein attachment to the surface is strongly affected by the change of water structure in this interface. Panos et al. [13] modeled protein–surface interactions to explore protein adsorption on polyurethane (PU) surfaces. The wettability of the membranes, as well as the surface roughness, strongly affected the total protein adsorption. Zhang et al. [14] found that the water layer structure determines the motion of atoms by guiding the electrostatic interactions, which explains why the adsorbed aqueous layers affect the adsorption dynamics so strongly. Therefore, water layers at the material interface define the relationship between the proteins and the material surface.

Surface-initiated atom transfer radical polymerization (SI-ATRP) is often used to graft polymeric brushes on material surfaces. Such surface modification maintains the original material structure, but improves its performance [15]. The attachment of zwitterions to the material surface improves the biocompatibility and antibacterial resistance of the membrane surfaces. However, they do not destroy bacteria, and small amounts of bacteria on the biofilm surfaces can still accumulate [16,17,18,19,20,21,22,23]. Liu et al. [24] reported a multi-step strategy for constructing poly-(sulfobetaine methacrylate) on thin-film-composite membranes via ATRP, followed by the in situ incorporation of silver nanoparticles (AgNPs). He et al. [25] designed an ATRP-based procedure to synthesize poly(sulfobetaine methacrylate) and quaternary ammonium salt. The resulting compound was then immobilized on a silicon wafer by a mussel-inspired chemical reaction. Yuan et al. [26,27] demonstrated a pathway of “repel and kill”. Poly(2-(dimethylamino)ethyl methacrylate) was integrated into polyethylene glycol terephthalate (PET) sheets and then quaternized by a ring-opening reaction to obtain polycarboxybetaine and polysulfobetaine. However, these reactions required a synergy of multiple ions or biocompatibility, which is only found in zwitterion-containing membranes.

Zwitterions are also difficult to use. Their counterparts—cationic polymers—also demonstrate high hydrophilicity and antibacterial properties [28,29,30]. Nowadays, cations play an essential role in biomedical applications. Metal, chitosan, and quaternary ammonium salt cations are among the most outstanding ones: PU films containing them show significantly enhanced hemocompatibility, anticoagulant, and antibacterial properties. Cai et al. [31] prepared an N-phosphonium chitosan with an excellent water solubility and tested it as an intramuscular vaccine delivery system using ovalbumin as a model antigen. Lei et al. [32] designed a new antibacterial thermoplastic polyurethane (TPU) nanofiber membrane by electrospinning copper-loaded NaX zeolite (CuX), which can effectively resist the adhesion of Escherichia coli (*E. coli*). Simultaneously, Cu^2+^ released by CuX in the nanofiber membrane destroys the cell wall and penetrates the plasma membrane of *Escherichia coli*, thus providing sterilization properties. Jin et al. [33] developed and applied a seminal surface-engineering strategy to conjugate anticoagulant heparin (Hep) and antibacterial carboxymethyl chitosan (CMCS) onto the surface of poly-dopamine (PDA)-coated PU film. Therefore, establishing a “kill and clear” strategy solely relying on cations is promising. Cationic brushes with antibacterial properties can be used to “kill” bacteria, and the consequent “clearing” step can be realized by a hydration layer. Catheter materials need a specific grafting density to achieve a dynamic balance of antibacterial and anticoagulant properties. However, research on cations capable of achieving this is still insufficient due to the underlying issues related to cytotoxicity and coagulability. Therefore, extensive qualitative analysis of various cations is desired.

To accomplish this, we used PU as a representative catheter material. Our early simulation work showed that grafting hydrophilic structures could negate interactions of the original substrate with the targeted proteins, stabilizing their natural conformation. Cationic grafting polymeric chains are inferior to zwitterionic and anionic ones, but are more effective than unmodified substrates. Because of the excellent antibacterial ability of cationic polymers, we further studied their grafting degree factor, especially with regards to the Acryl-oxy-ethyl-trimethyl-ammonium chloride (DAC) structure and the natural protein conformations. PU was functionalized with cationic polymer brushes using SI-ATRP to obtain a material capable of a “kill and clear” approach. We also attempted to optimize the PU grafting density using hemocompatibility experiments for guidance. Our experimental and theoretical results provide insights and novel ideas for advancing research and the development of materials for catheters.

## 2. Theoretical Calculations

**Figure 1:**
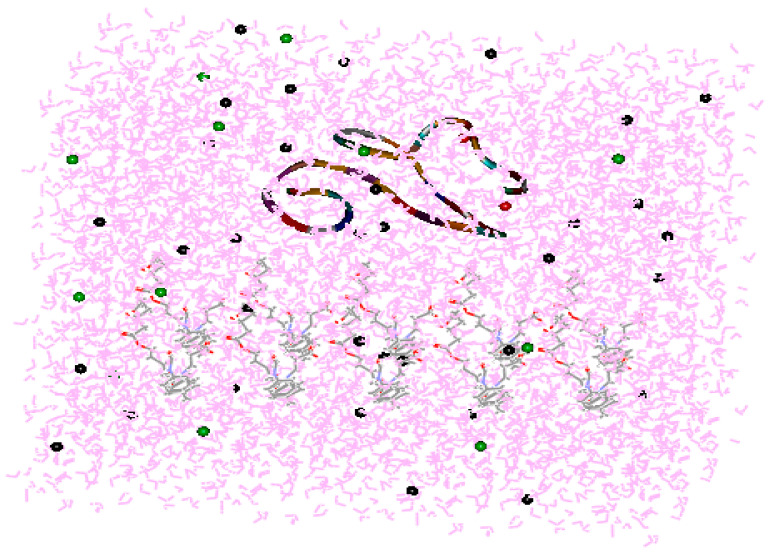
Model showing interactions between the calcium-binding epidermal growth factor (cbEGF)-like domain and polyurethane (PU) in the presence of water. The red dots represent Ca^2+^ complexed with an EGF-like domain. The green dots are Cl^−^. The black dots are Na^+^. Pink represents water molecules.

In this study, 20, 40, 60, and 80% grafting degrees were achieved by adjusting the PU and DAC-g-PU chain ratios. The calcium-binding epidermal growth factor-like-domain (cbEFG-like-domain) of coagulation factor IX was chosen as a research object, similar to our previous work. Chemistry at HARvard Macromolecular mechanics (CHARMm) force field and Simple Point Charge (SPC) solvent models were used (Figure 1). After energy minimization and system equilibration, 10 ns molecular dynamics (MD) simulations (with a 2 fs step size) were conducted in isothermal-isobaric ensemble (NPT) at 310 K and 1 atmfor each grafting percentage [34,35,36,37].

## 3. Experimental Section

### 3.1. Materials

Medical-grade PU particles were provided by Nanjing Xianbang Co. (Shanghai, China). Toluene (AR), N, N-dimethylformamide (DMF, AR), triethylamine (TEA, 99%), methanol (AR), anhydrous ether (AR), and 2,2-bipyridine (BPY, 99.5%) were obtained from Sinopharm Chemical Reagent Co. (Shanghai, China). Copper (I) bromide (98.5%), 4,4-diphenylmethane diisocyanate (MDI, AR), 2-bromoethanol (96%), and dimethyl-diallyl-ammonium chloride (DMDAAC, 70%) were purchased from Shanghai Aladdin Bio-Chem Technology (Shanghai, China). Methacryl-oxy-oxyethyl-trimethyl-ammonium chloride (DMC, 97%) was provided by Shanghai Macklin Bio-Chem Technology Co. (Shanghai, China). Acryl-oxy-ethyl-trimethyl-ammonium chloride (DAC, 80%) was purchased from Sigma-Aldrich (Shanghai, China). Plasma enriched with platelets and fresh human blood was obtained from the Nanjing Gulou hospital (Nanjing, China). The BCA protein quantification kit was also purchased from Sigma-Aldrich (Shanghai, China).

### 3.2. Preparation of PU Films

The PU particles were mixed with DMF to obtain an 8 wt% mixture, which was then transferred to a round Teflon disc (10 cm in diameter) for evaporation, conducted in vacuum at 50 °C for 48 h. The resulting PU films were extracted with toluene and ethanol using a Soxhlet extractor for 12 h. The samples were dried and stored in a vacuum.

### 3.3. Immobilization of Initiator on PU Films

For PU surface activation, 4 g of MDI was dissolved in 40 mL of toluene under constant stirring. The resulting solution was sealed under nitrogen. A total of 0.5 mL of TEA was added to act as a catalyst. The PU films were then heated to 60 °C for 1 h under nitrogen. The resulting PU samples (marked as PU-NCO) were soaked in toluene for 20 h, followed by vacuum-drying at 40 °C for 12 h.

The activated surface groups were converted into initiators by soaking PU-NCO samples in a solution containing 3 mL of 2-bromoethanol and 30 mL of anhydrous ether. Following this, 0.5 mL of TEA was added under nitrogen. The mixture was allowed to react for 10, 20, 30, 40, and 60 min at room temperature. The resulting PU samples (marked as PU-Br) were soaked in anhydrous ether for 15 h and vacuum-dried at 40 °C for 5 h.

### 3.4. SI-ATRP from PU Films

The functionalization of PU films was performed by SI-ATRP based on the procedure described in our previous work [38]. For this purpose, 1.0 mmol of BPY and several pieces of PU-Br were added to a Schlenk flask, which was then evacuated using liquid nitrogen cryotherapy and back-filled with nitrogen. Following this, 0.5 mmol of CuBr, 4.0 mmol of cation solution, and 20 mL of 1:1 (by volume) methanol/water mixture were degassed and then added to the flask under nitrogen. The mixture was stirred at room temperature for 2 h polymerization, after which the samples were sonicated in PBS and water (for 10 min in each) and vacuum-dried at 40 °C for 12 h. The samples obtained in this step were marked as PU-X and PU-Y, where X is the reaction time and Y is the cation type.

### 3.5. Characterization

Fourier transform infrared (FTIR) spectra were recorded using an ALPHA II instrument manufactured by BRUKER (Karlsruhe, Germany) in the 4000–500 cm^−1^ range. Each spectrum was obtained after 64 scans at a 4 cm^−1^ resolution. X-ray photoelectron spectra (XPS) were collected using an ESCALAB Xi+ instrument manufactured by Thermo Scientific (Waltham, MA, USA) employing Al Kα radiation as a source and the C1s hydrocarbon peak at 284.6 eV as a reference. The water contact angle (WCA) measurements were performed using a DSA30S instrument manufactured by KRUSS (Hamburg, Germany). Furthermore, 3 µL water droplets on the material surfaces were recorded by a video camera to analyze their contact angles.

### 3.6. Protein Adsorption of PU

To conduct protein adsorption experiments, the samples were first soaked in PBS solution overnight and then in 2 mL of platelet-poor plasma (PPP) made from platelet-rich plasma, and were then incubated at 37 °C for 90 min, after which the samples were rinsed three times in PBS. The adsorbed proteins were separated by 20 min ultrasonication in the SDS solution. The adsorbed protein content was determined by the BCA protein quantification kit, using a standard protein solution as the calibration standard [39,40].

### 3.7. Hemolysis Assays of PU

PU samples were washed with PBS and soaked in 0.9% saline solution overnight. Diluted blood was prepared by blending 2 mL of fresh human whole blood (containing sodium citrate) and 2.5 mL of 0.9% saline solution. Subsequently, 0.2 mL of diluted blood was added to the saline solution containing PU samples and incubated at 37 °C for 120 min, after which it was centrifuged at 1000 rpm for 10 min. The supernatant was transferred to a cuvette for optical density (O.D.) evaluation performed under 545 nm by a Multiskan GO microplate reader manufactured by Thermo Scientific (USA). The positive and negative controls were diluted with distilled water and 0.9% NaCl aqueous solution, respectively, to increase the blood volume to 10 mL. The controls were then shaken for 1 h and centrifuged at 1000 rpm for 10 min. The supernatant was used for O.D. analysis at 545 nm [41,42].

The hemolysis rate was calculated using the following formula:

$$\mathrm{Hemolysis}\;\mathrm{rate}\left(\%\right)=\frac{D_{t}-D_{nc}}{D_{pc}-D_{nc}}\times 100\%,$$

where $D_{t}$ is the sample absorbance, and $D_{nc}$ and $D_{pc}$ are absorbance values of the negative and positive controls, respectively.

### 3.8. Anticoagulant Measurements of PU In Vitro

After overnight equilibration in PBS, the samples were added to a 96-well tissue culture plate. Then, 1 mL of PPP and 1 mL of 0.025 mol/L CaCl_2_ solutions were added to each well. The recalcification time required for flocculent precipitation was recorded. The O.D. was analyzed at 405 nm. The time stopped when the curve had an inflection point [42].

### 3.9. E. coli Adhesion to PU

The liquid culture medium used for bacteria was sterilized in an autoclave for 120 min and then added to a 24-well tissue culture plate at 37 °C. Next, 10 μm of *E. coli* toxin was added to each well. The PU samples, equilibrated with PBS overnight, were placed in a 24-well plate and incubated at 37 °C for 24 h, after which the samples were washed with PBS to remove non-adherent bacteria, fixed with a 2.5 wt% glutaraldehyde aqueous solution, and stored at 4 °C for 4 h. The resulting samples were soaked in several ethanol/distilled water solutions (1:1, 3:2, 7:3, 4:1, and 5:0 (by volume) in sequence, 15 min for each solution) for dehydration. The resulting samples were freeze-dried, sprayed with gold, and analyzed for adhered bacteria using a JSM-5610LV scanning electron microscope (SEM) by JEOL (Tokyo, Japan) [26,40].

## 4. Results and Discussion

### 4.1. The Intermolecular Hydrogen Bonds

**Figure 2:**
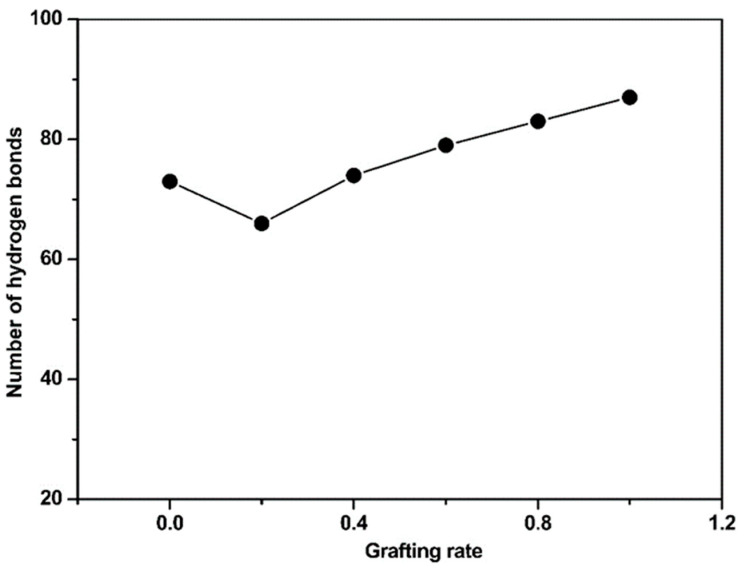
Number of hydrogen bonds in the cbEGF-like domain-acryl-oxy-ethyl-trimethyl-ammonium chloride (DAC)-g-PU surface as a function of the grafting degree.

Our previous simulations demonstrated that grafting hydrophilic structures could minimize the effects of the original substrate surfaces on targeted proteins by stabilizing their natural conformations. In this work, we mainly focused on the hydrogen bonding between the grafted surfaces and the protein conformations. At grafting rates above 40%, the H-bond content was linear (see Figure 2). At a 20% grafting degree, the EGF-like domain contained the lowest number of H-bonds with the grafted polymer. Such an abnormally low content is related to the specific properties of the DAC-grafted surface and its interaction with a protein. Therefore, grafting degree optimization will result in the most suitable biocompatibility and antibacterial ability of the cationically grafted polymers.

### 4.2. PU Grafting Density

The PU grafting density can be expressed as follows [43]:

$$\mathrm{Grafting}\;\mathrm{Density}=\frac{W_{g}-W_{0}}{s},$$

where $W_{g}$ is the sample weight after grafting, and $W_{0}$ is the PU-Br sample weight. The grafting densities of the various cationic PU samples are listed in Table 1. The grafting density gradually increased as the immobilization time increased. The highest grafting density equal to 167 μg/cm^2^ was achieved after a 60 min immobilization time.

### 4.3. Characterization of Functionalized PU Samples

**Figure 3:**
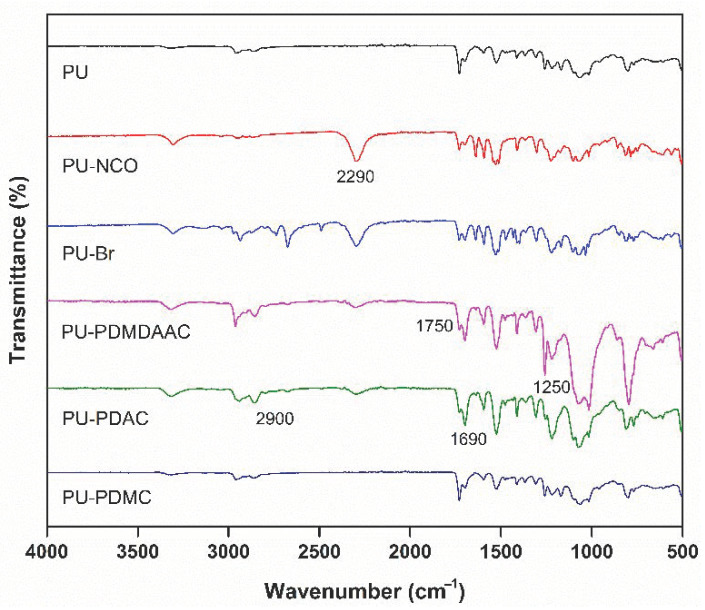
Fourier transform infrared (FTIR) spectra of pure and modified PU prepared in this work.

The FTIR spectra of PU-NCO samples showed a strong band at 2290 cm^−1^ corresponding to the NCO stretching vibrations (see Figure 3). This band was absent in the FTIR spectra of pure PU. Absorbance at 2290 cm^−1^ became weaker after immobilization by Br [38]. A peak corresponding to –C=O group PU-PDMDAAC appeared at 1750 cm^−1^, while a peak corresponding to –CH_3_ [44] was detected at 1250 cm^−1^. Methyl asymmetric stretching vibrations are characterized by absorption in the 2800–2900 cm^−1^ region. –C=O bonds of PU-PDAC manifested themselves by an absorption band at 1690 cm^−1^ [45].

**Figure 4:**
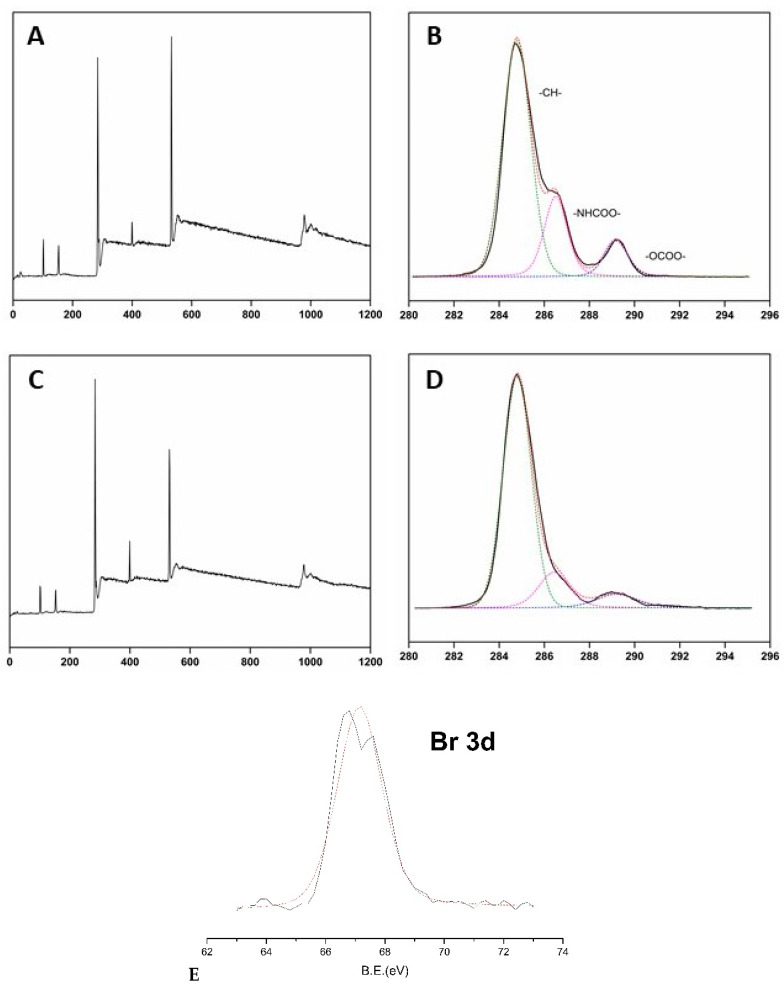
X-ray photoelectron spectra (XPS) survey (**A**,**C**) and high-resolution C1s (**B**,**D**,**E**) spectra of PU (**A**,**B**) and PU-Br (**C**,**D**,**E**) samples.

The XPS survey spectrum of unmodified PU showed peaks at 285, 533, and 400 eV (see Figure 4A), which belonged to C, O, and N binding energies, respectively [43]. PU-Br samples displayed additional peaks at 68 and 188 eV, which were attributed to Br 3d and Br 3p binding energies, respectively (see Figure 4C) [38]. The XPS spectrum of cationically grafted PU samples did not exhibit peaks belonging to –OCOO–, –HNCOO–, –CH–, and –C–Br groups, which were present before grafting. The carbon XPS peak in the spectrum of grafted PU had a different binding energy than in the spectra of PU and PU-Br samples because of the presence of C– and O– containing cations (DAC and DMC). The C/O ratio also changed because of the PU modification by grafting due to the substitution of several carbon atoms by O and N. The C/O ratio of unmodified PU (which was equal to 2.61) increased, confirming the successful polymerization of cationic brushes based on PU (see Table 2).

**Figure 5:**
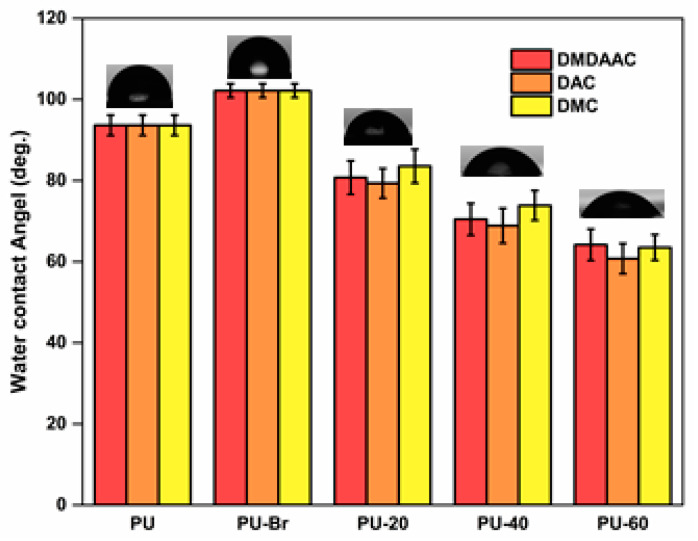
Water contact angle (WCA) data for various PU samples.

WCA tests showed that PU was hydrophobic, judging by its WCA of 93° (see Figure 5) [46,47]. After the immobilization, WCA increased to 102° because of the presence of a more hydrophobic Br– containing initiator [43]. Grafting PU with cationic brushes resulted in a significantly reduced WCA. Therefore, grafted PU became hydrophilic because of the presence of hydrophilic functional groups on the ends of the molecules of the cationic brushes [48]. Additionally, the WCA decreased as the grafting degree increased.

### 4.4. Protein Adsorption

**Figure 6:**
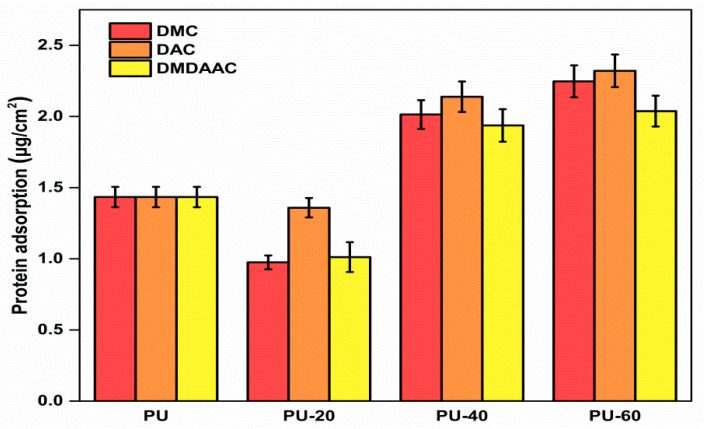
Protein adsorption by PU grafted with methacryl-oxy-oxyethyl-trimethyl-ammonium chloride (DMC), DAC, and DMDAAC canyon brushes possessing different grafting densities (20, 40, and 60%). The error bars represent the standard deviation from the average value calculated using three measurements.

The amount of protein adsorption on unmodified PU after 90 min incubation in fresh PPP was 1.434 μg/cm^2^ (see Figure 6), which is higher than for other hydrophilic materials [49,50]. Such a high value could be explained by the PU hydrophobicity [51], which does not allow hydrophilic proteins to bind to its surface. After the grafting with cationic brushes, the amount of adsorbed protein decreased for materials with low grafting degrees and increased for materials with high grafting densities. The PU-20 sample demonstrated the most optimal protein adsorption amount. Negatively charged blood proteins were somewhat attracted to the slightly grafted PU materials because the electrostatic effect was weak. We believe that the primary mechanism responsible for protein attraction is related to the surface hydrophilicity. Hydrophilic surfaces can better accommodate the natural states of the adsorbed proteins. Protein serums of the slightly alkaline human blood serum are negatively charged. Therefore, their structures are expected to be dependent on their electrostatic adsorption at the material surface. Additionally, the higher the surface density, the more protein will be adsorbed by the cationically grafted PU polymers [52].

### 4.5. Hemocompatibility In Vitro

**Figure 7:**
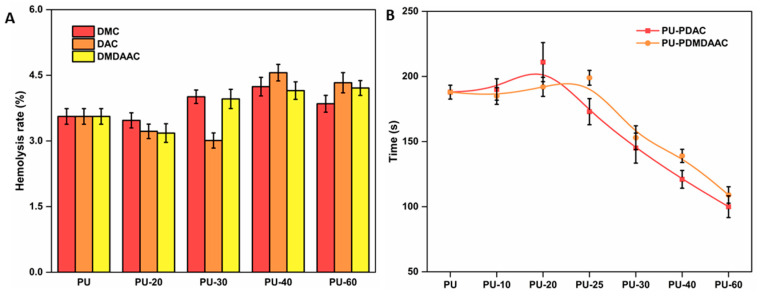
(**A**) Hemolysis rate of the PU grafted with DMC, DAC, and DMDAAC cationic brushes and possessing different grafting densities. (**B**) Recalcification time of cationic brushes possessing different grafting densities. The values represent the average of three measurements. The error bars are standard deviations from the average.

Catheter material compatibility with blood is essential for patients’ well-being [40,53]. The anti-hemolytic ability of PU grafted with cationic brushes increased at a low grafting density. However, the hemolysis rates of these samples were higher at higher grafting densities (see Figure 7A). Cationic surfactants with strong bactericidal and hemolytic properties are often added during pesticide manufacturing. The surfaces of the cells in human blood are negatively charged, which results in their strong electrostatic attraction to a positively charged material surface. The molecular chain on the material surface inserts itself into the blood cell membrane, changing its osmotic pressure and causing a rupture. PU samples functionalized with different cationic brushes showed very similar hemocompatibility ranges, with the most optimum value when the reaction time was 25 min.

Soluble fibrinogen is converted into soluble fibrin by adding Ca^2+^ to platelet plasma and cross-linking its proteins with the thrombus [54,55]. The longer the recalcification time, the better the anticoagulant effect. The recalcification times obtained in this work for the grafted PU displayed the same trend as the protein adsorption (see Figure 7B). The recalcification time was longer for material with a lower grafting density. These results also indicate that these materials possessed a coagulation ability, which was enhanced even more for high grafting density materials. Although PU grafted with different cations demonstrated different anticoagulant times, the best anticoagulation effect was achieved for samples with a 20% grafting degree.

### 4.6. E. coli Adhesion

**Figure 8:**
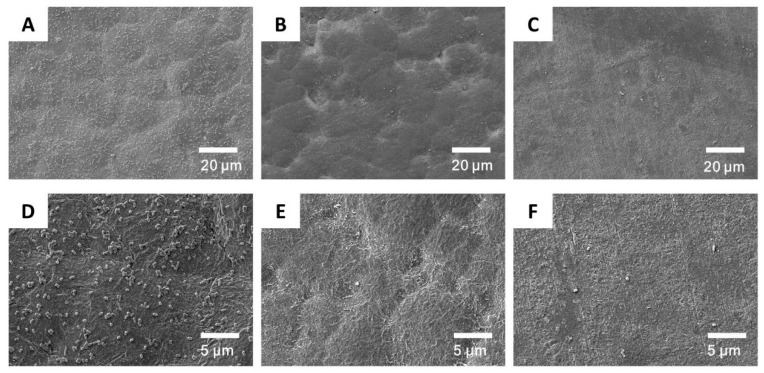
Scanning electron microscope (SEM) micrographs of bacteria accumulation on PU (**A**,**D**), PU-PDAC (**B**,**E**), and PU-PDMDAAC (**C**,**F**).

Biofilms form on the catheter surface due to bacterial adhesion [56]. This film protects bacteria from human immune system attacks [57]. The inhibition of biofilm formation by killing bacteria could prevent the infection. Figure 8 shows the adsorption of *E. coli* on unmodified PU and grafted PU-20 materials. Numerous bacteria were visible on the unmodified PU surfaces. However, significantly fewer bacteria were detected on the grafted PU surface because of the presence of cationic brushes, which interact with the negatively charged bacterial cell membranes, disrupting their cytoplasmic membrane and causing cell lysis. Therefore, PU grafted with cationic brushes demonstrated excellent antibacterial properties. The most optimal results were obtained for samples subjected to a 20 min grafting procedure.

### 4.7. Zeta Potential

**Figure 9:**
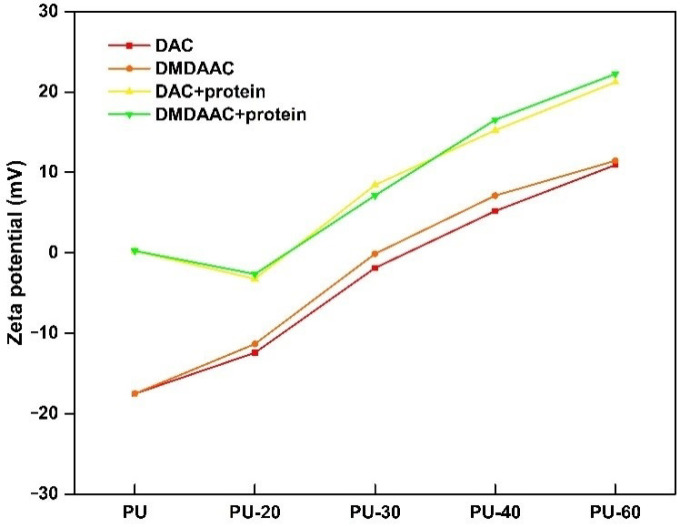
Zeta potential in the water of different PU composites.

The zeta potential of the unmodified PU was very low (−17.52, see Figure 9), which was attributed to the presence of –OH and –COOH groups on the PU surface. After grafting, the zeta potential increased as a function of the grafting density and eventually became positive when the grafting density was 133 μg/cm^2^ (PU-40). An analysis of the zeta potential changes during and after the protein adsorption experiment revealed that the zeta potential could be used to judge the protein adsorption degree. Additionally, we observed that the protein adsorption by the modified PU depends on its grafting density.

## 5. Conclusions

This work reported a strategy of constructing functionalized anti-biofilms containing cationic brushes with antifouling and bactericidal properties. The cationic polymeric brushes were synthesized using the SI-ATRP method and PU surface as a substrate. The optimum grafting density, which resulted in the best hemocompatibility and anti-biofouling ability, was equal to 86 μg/cm^2^. Our theoretical simulations verified that the optimal hemocompatibility could be obtained at a 20% grafting degree. We also provided insights into the mechanism responsible for the excellent biocompatibility of PU substrates modified with cationic brushes. Negatively charged blood proteins remained in their natural states during their interaction with the hydrophilic surface of the grafted PU. As the grafting degree of PU surface was increased, the electrostatic effect increased, which, in turn, reduced the hemocompatibility of these materials. The materials developed in this work demonstrate a novel and promising (for practical applications) concept for obtaining antimicrobial and biocompatible surfaces for medical catheters.

## Figures and Tables

**Table 1:** Graft density of PU samples.

Sample ID	Graft Density, μg/cm^2^
PU-10	66
PU-20	86
PU-30	112
PU-40	133
PU-60	167

**Table 2:** Results of the XPS analysis of the PU sample surfaces prepared in this work.

Sample ID	Element Content, at%				
	C	O	N	Br	C/O
PU	70.79	27.04	1.78	/	2.61
PU-NCO	78.75	10.06	11.19	/	7.82
PU-Br	76.08	14.84	8.32	0.76	5.13
PU-PDMC	69.35	24.91	5.74	/	2.78
PU-PDAC	69.76	23.26	6.83	0.05	3.00
PU-PDMDAAC	69.41	24.33	6.19	0.07	2.85

## References

[1] Rickard C.M., Nicole M., Joan W., Runnegar N., Larsen E., McGrail M.R., Fullerton F., Bettington E., Whitty J.A., Choudhury M.A. (2018). Dressings and securements for the prevention of peripheral intravenous catheter failure in adults (SAVE): A pragmatic, randomised controlled, superiority trial. Lancet.

[2] Ariza H., Ella J. (2018). Update on infection control practices in cancer hospitals. CA Cancer J. Clin..

[3] Laura L. (2019). Antibiotic locks for the treatment of catheter-related blood stream infection: Still more hope than data. Semin. Dial..

[4] Liang W., Wei F., Yang C. (2020). GDF-15 is associated with thrombus burden in patients with deep venous thrombosis. Thromb. Res..

[5] Keum H., Kim J.Y., Yu B., Yu S.J., Kim J., Jeon H., Lee D.Y., Im S.G., Jon S. (2017). Prevention of bacterial colonization on catheters by a one-step coating process involving an antibiofouling Polymer in Water. ACS Appl. Mater. Interfaces.

[6] Riool M., de Breij A., Drijfhout J.W., Nibbering P.H., Zaat S.A. (2017). Antimicrobial peptides in biomedical device manufacturing. Front. Chem..

[7] Ekdahl K.N., Soveri I., Hilborn J., Fellström B., Nilsson B. (2017). Cardiovascular disease in haemodialysis: Role of the intravascular innate immune system. Nat. Rev. Nephrol..

[8] Murray J., Precious E., Alikhan R. (2013). Catheter-related thrombosis in cancer patients. Br. J. Haematol..

[9] Yamada S., Motozuka S., Tagaya M. (2020). Synthesis of nanostructured silica/hydroxyapatite hybrid particles containing amphiphilic triblock copolymer for effectively controlling hydration layer structures with cytocompatibility. J. Mater. Chem. B.

[10] Adamczyk Z. (2019). Protein adsorption: A quest for a universal mechanism. Curr. Opin. Colloid Interface Sci..

[11] Stepniewski M., Pasenkiewicz-Gierula M., Ro G.T., Danne R., Orlowski A., Karttunen M., Urtti A., Yliperttula M., Vuorimaa E., Bunker A. (2011). Study of PEGylated lipid layers as a model for PEGylated liposome surfaces: Molecular dynamics simulation and Langmuir monolayer studies. Langmuir.

[12] Cormack A.N., Lewis R.J., Goldstein A.H. (2004). Computer simulation of protein adsorption to a material surface in aqueous solution: Biomaterials modeling of a ternary system. J. Phys. Chem. B.

[13] Panos M., Sen T.Z., Ahunbay M.G. (2012). Molecular simulation of fibronectin adsorption onto polyurethane surfaces. Langmuir.

[14] Zhang C., Li X., Wang Z., Huang X., Ge Z., Hu B. (2020). Influence of structured water layers on protein adsorption process: A case study of cytochrome c and carbon nanotube interactions and its implications. J. Phys. Chem. B.

[15] Wang M., Wang X., Zhang K., Wu M.Y., Wu Q.Y., Liu J.Y., Yang J.J., Zhang J.N. (2020). Nano-hydroxyapatite particle brushes via direct initiator tethering and surface-initiated atom transfer radical polymerization for dual responsive Pickering emulsion. Langmuir.

[16] Meng F., Zhang M., Ding K. (2018). Cell membrane mimetic PVDF microfiltration membrane with enhanced antifouling and separation performance for oil/water mixtures. J. Mater. Chem. A.

[17] Debayle M., Balloul E., Dembele F., Xu X., Hanafi M., Ribot F., Monzel C., Coppey M., Fragola A., Dahan M. (2019). Zwitterionic polymer ligands: An ideal surface coating to totally suppress protein-nanoparticle corona formation. Biomaterials.

[18] Zhang C., Yin C., Wang Y. (2020). Simultaneous zwitterionization and selective swelling-induced pore generation of block copolymers for antifouling ultrafiltration membranes. J. Membr. Sci..

[19] Pullanchery S., Yang T., Cremer P.S. (2018). Introduction of positive charges into zwitterionic phospholipid monolayers disrupts water structure whereas negative charges enhances it. J. Phys. Chem. B.

[20] Zhang Y., Liu Y., Ren B. (2019). Fundamentals and applications of zwitterionic antifouling polymers. J. Phys. D Appl. Phys..

[21] Tsai M.C., Hung K.C., Hung S.C., Hsu S.H. (2015). Evaluation of biodegradable elastic scaffolds made of anionic polyurethane for cartilage tissue engineering. Colloids Surf. B Biointerfaces.

[22] Zhang H., Chiao M. (2015). Anti-fouling coatings of poly (dimethylsiloxane) devices for biological and biomedical applications. J. Med. Biol. Eng..

[23] Tasia W., Lei C., Cao Y., Ye Q., He Y., Xu C. (2020). Enhanced eradication of bacterial biofilms with DNase I-loaded silver-doped mesoporous silica nanoparticles. Nanoscale.

[24] Liu C., Faria A.F., Ma J., Elimelech M. (2017). Mitigation of Biofilm Development on Thin-Film Composite Membranes Functionalized with Zwitterionic Polymers and Silver Nanoparticles. Environ. Sci. Technol..

[25] He Y., Wan X., Xiao K., Lin W., Li J., Li Z., Luo F., Tan H., Li J., Fu Q. (2019). Anti-biofilm surfaces from mixed dopamine modified polymer brushes: Synergistic role of cationic and zwitterionic chains to resist staphylococcus aureus. Biomater. Sci..

[26] Wang Y., Shen J., Yuan J. (2016). Design of hemocompatible and antifouling PET sheets with synergistic zwitterionic surfaces. J. Colloid Interface Sci..

[27] Jin X., Yuan J., Shen J. (2016). Zwitterionic polymer brushes via dopamine-initiated ATRP from PET sheets for improving hemocompatible and antifouling properties. Colloids Surf. B Biointerfaces.

[28] Robinson D.A., Griffith R.W., Shechtman D., Evans R.B., Conzemius M.G. (2010). In vitro antibacterial properties of magnesium metal against Escherichia coli, Pseudomonas aeruginosa and Staphylococcus aureus. Acta Biomater..

[29] Li P., Poon Y.F., Li W., Zhu H.Y., Yeap S.H., Cao Y., Qi X., Zhou C., Lamrani M., Beuerman R.W. (2010). A polycationic antimicrobial and biocompatible hydrogel with microbe membrane suctioning ability. Nat. Mater..

[30] Gao D., Feng J., Ma J. (2014). Synthesis of cationic binder through surfactant-free emulsion polymerization for textile pigment applications. Prog. Org. Coat..

[31] Cai J., Zhang W., Xu J., Xue W., Liu Z. (2017). Evaluation of N-phosphonium chitosan as a novel vaccine carrier for intramuscular immunization. J. Biomater. Appl..

[32] Lei J., Yao G., Sun Z., Wang B., Yu C., Zheng S. (2019). Fabrication of a novel antibacterial TPU nanofiber membrane containing Cu-loaded zeolite and its antibacterial activity toward Escherichia coli. J. Mater. Sci..

[33] Jin Y., Zhu Z., Liang L., Lan K., Zheng Q., Wang Y., Guo Y., Zhu K., Mehmood R., Wang B. (2020). A facile heparin/carboxymethyl chitosan coating mediated by polydopamine on implants for hemocompatibility and antibacterial properties. Appl. Surf. Sci..

[34] Brooks B.R., Brooks C.L., Mackerell A.D., Nilsson L., Petrella R.J., Roux B., Won Y., Archontis G., Bartels C., Boresch S. (2009). CHARMm: The biomolecular simulation program. J. Comput. Chem..

[35] Vanommeslaeghe K., Hatcher E., Acharya C., Kundu S., Zhong S., Shim J., Darian E., Guvench O., Lopes P., Vorobyov I. (2010). CHARMm general force field: A force field for drug-like molecules compatible with the CHARMm all-atom additive biological force fields. J. Comput. Chem..

[36] Choi W., Jin J., Park S., Kim J.Y., Lee M.J., Sun H., Kwon J.S., Lee H., Choi S.H., Hong J. (2020). Quantitative interpretation of hydration dynamics enabled the fabrication of a zwitterionic antifouling surface. ACS Appl. Mater. Interfaces.

[37] Walke C.D., Chan W.C. (2012). Understanding and controlling the interaction of nanomaterials with proteins in a physiological environment. Chem. Soc. Rev..

[38] Liu P., Huang T., Liu P., Shi S., Chen Q., Li L., Shen J. (2016). Zwitterionic modification of polyurethane membranes for enhancing the anti-fouling property. J. Colloid Interface Sci..

[39] Wang H., Hu Y., Lynch D., Young M., Li S., Cong H., Xu F.J., Cheng G. (2018). Zwitterionic Polyurethanes with Tunable Surface and Bulk Properties. ACS Appl. Mater. Interfaces.

[40] Zhang X., Zhao Y., Zhang Y., Wang A., Ding X., Li Y., Duan S., Ding X., Xu F.J. (2019). Antimicrobial Peptide-Conjugated Hierarchical Antifouling Polymer Brushes for Functionalized Catheter Surfaces. Biomacromolecules.

[41] Shitole A.A., Raut P.W., Khandwekar A. (2019). Design and engineering ofpolyvinyl alcohol based biomimetic hydrogels for wound healing and repair. J. Polym. Res..

[42] Zhang J., Liu C., Feng F., Wang D., Lu S.S., Wei M.H., Qiao T.G. (2017). A PC–PU nanoparticle/PU/decellularized scaffold composite vascular patch: Synergistically optimized overall performance promotes endothelialization. Colloids Surf. B Biointerfaces.

[43] Yuan H., Qian B., Zhang W. (2016). Protein adsorption resistance of PVP-modified polyurethane film prepared by surface-initiated atom transfer radical polymerization. Appl. Surf. Sci..

[44] Xu Q., Peng J., Zhang W. (2020). Electrospun cellulose acetate/P(DMDAAC-AM) nanofibrous membranes for dye adsorption. J. Appl. Polym. Sci..

[45] Wang H., Zhang Y., Gao D. (2019). Research on self-degradation of RGO/TiO2-P(AM-DAC) organic-inorganic composite flocculant prepared by surface initiated polymerization and its flocculation mechanism of oil sand tailings. Eur. Polym. J..

[46] Chua S.C., Chong F.K., Ul Mustafa M.R., Mohamed S.R., Sujarwo W., Abdul M.M., Show P.L., Ho Y.C. (2020). Microwave radiation-induced grafting of 2-methacryloyloxyethyl trimethyl ammonium chloride onto lentil extract (LE-g-DMC) as an emerging high-performance plant-based grafted coagulant. Sci. Rep..

[47] Wang G., Liu Z., Zhang N. (2018). Super hydrophilicity of hydroxy modified poly (M-Phenylenediamine) aerogel for separation of oil/water and biocompatibility. Mater. Res. Express..

[48] Hoang B.N., Nguyen T.T., Bui Q.P.T. (2019). Enhanced selective adsorption of cation organic dyes on polyvinyl alcohol/agar/maltodextrin water-resistance biomembrane. J. Appl. Polym. Sci..

[49] Lien C.C., Chen P.J., Venault A. (2019). Zwitterionic interpenetrating network for improving the blood compatibility of polypropylene membranes applied to leukodepletion. J. Membr. Sci..

[50] Liu P., Chen Q., Liu X., Yuan B., Wu S.S., Shen J., Lin S.C. (2009). Grafting of zwitterion from cellulose membranes via ATRP for improving blood compatibility. Biomacromolecules.

[51] Beilis E., Belgorodsky B., Fadeev L., Cohen H., Richter S. (2014). Surface-induced conformational changes in doped bovine serum albumin self-assembled monolayers. J. Am. Chem. Soc..

[52] Wang L., Zhu M., Miao R. (2017). Effect of monovalent cations on ultrafiltration membrane fouling of protein. China Environ. Sci..

[53] Refaai M.A., Phipps R.P., Spinelli S.L., Blumberg N. (2011). Platelet transfusions: Impact on hemostasis, thrombosis, inflammation and clinical outcomes. Thromb. Res..

[54] Fu X., Ning J.P. (2018). Synthesis and biocompatibility of an argatroban-modified polysulfone membrane that directly inhibits thrombosis. J. Mater. Sci. Mater. Med..

[55] Chi C., Sun B., Zhou N., Zhang M., Chu X., Yuan P., Shen J. (2018). Anticoagulant polyurethane substrates modified with poly (2-methacryloyloxyethyl phosphorylcholine) via SI-RATRP. Colloids Surf. B Biointerfaces.

[56] Habimana O., Semiao A.J.C., Casey E. (2014). The role of cell-surface interactions in bacterial initial adhesion and consequent biofilm formation on nanofiltration/reverse osmosis membranes. J. Membr. Sci..

[57] Burton E.A., Simon K.A., Hou S., Ren D., Luk Y.Y. (2009). Molecular gradients of bioinertness reveal a mechanistic difference between mammalian cell adhesion and bacterial biofilm formation. Langmuir.

